# Supplementary material for: Placental miR-340 mediates vulnerability to activity based anorexia in mice
Source: Nat Commun. 2018 Apr 23;9:1596. doi: 10.1038/s41467-018-03836-2 (PMC5913294; doi:10.1038/s41467-018-03836-2)
Supplement: Supplementary file 1 — Supplementary Information [file 41467_2018_3836_MOESM1_ESM.pdf]

Supplementary Fig. 1

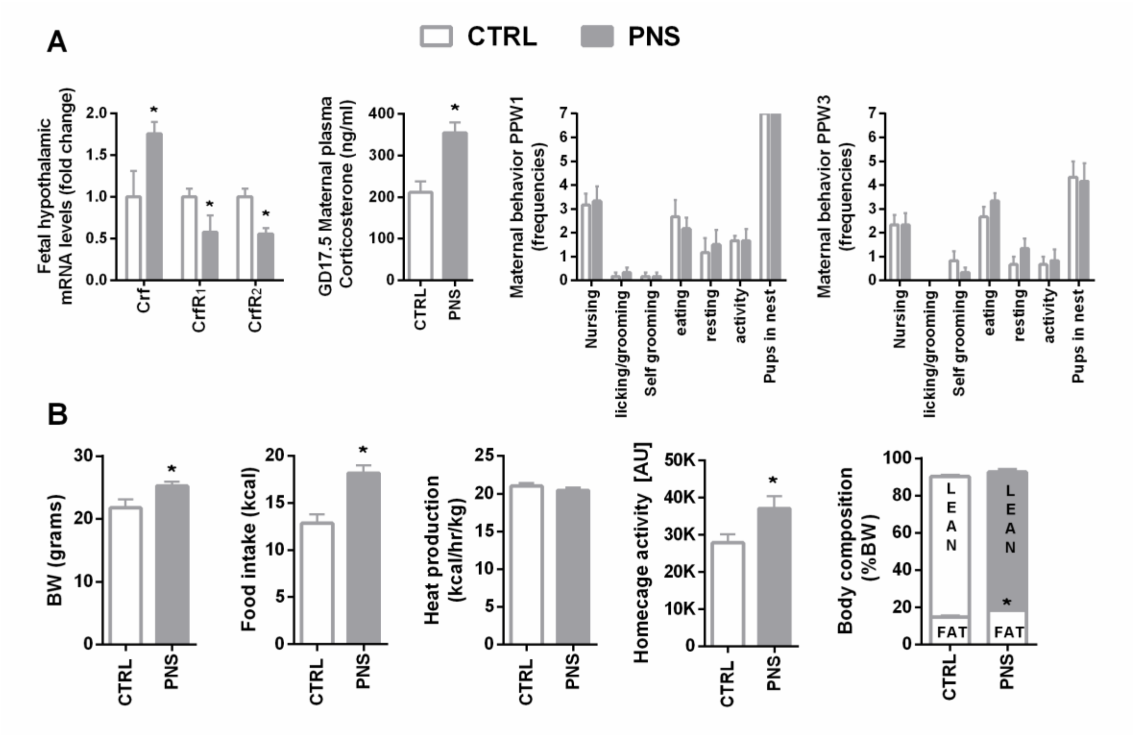

Supplementary Fig. 1 Prenatal stress induced stress related responses in the mother and the fetuses. (A) The CRF system is dysregulates in female fetuses (Two-way ANOVA $F_{int(2,30)}$=7.66, p=0.002). PNS increase plasma CORT in the mother ($t_{(10)}$=3.88, p=0.003). Maternal behavior was not affected was not affected by the manipulation. **(B)** PNS affected basal metabolism in females by increasing BW ($t_{(10)}$=2,32, p=0.043), food intake ($t_{(10)}$=4,32, p=0.0015), activity ($t_{(10)}$=2,25, p=0.0478) and body fat ($t_{(10)}$=2,45, p=0.034) but not heat production. Data presented in mean and SEM.

Supplementary Fig. 2

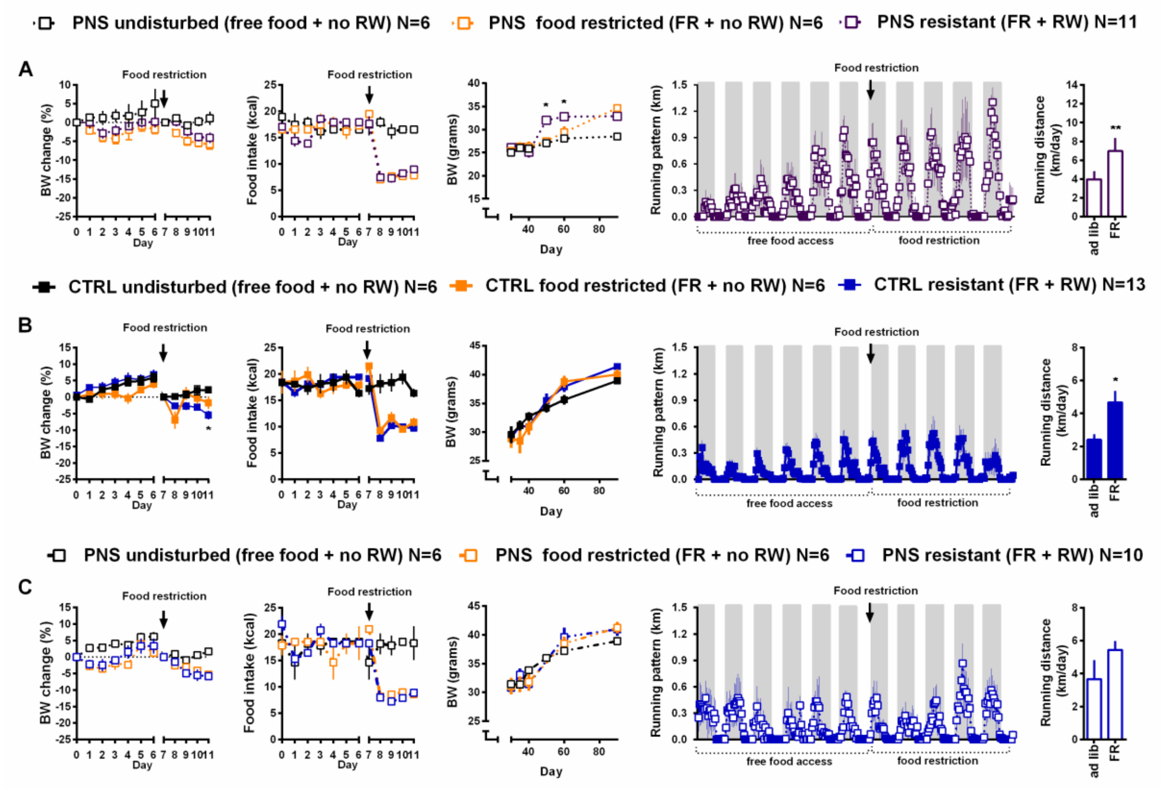

Supplementary Fig. 2 Activity based anorexia (ABA) vulnerability was abolished in adolescent PNS females **(A)**, in adolescent CTRL males **(B)** and in adolescent PNS males **(C)**. Specifically, the ABA protocol didn't produce perceivable changes in BW or food intake in any of the groups, when compared to food restricted controls. Despite a transient increase during the recovery phase, long term BW was not affected by the ABA protocol. Running pattern was normal in and total running distance during FR was moderately increased. Data presented in mean and SEM. CTRL: control. RW: running wheel.

Supplementary Fig. 3

A

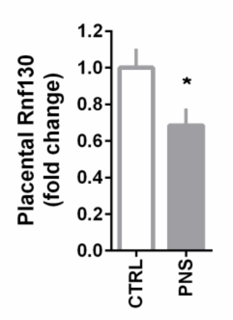

B

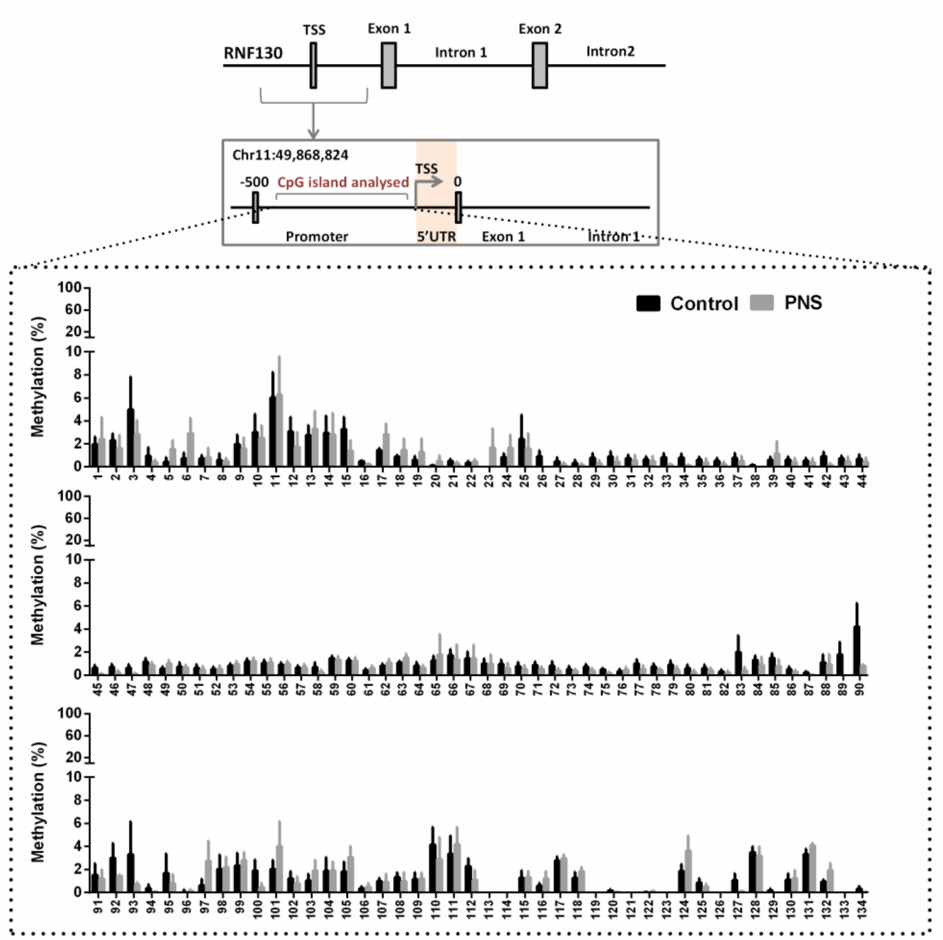

Supplementary Fig. 3 (A) Rfn130 gene expression was reduced by PNS ($t_{(21)}$=2.262, p=0.034, N=11,12). **(B)** Rnf130 promoter DNA methylation analysis showed no differences between controls and PNS.

Supplementary Fig. 4

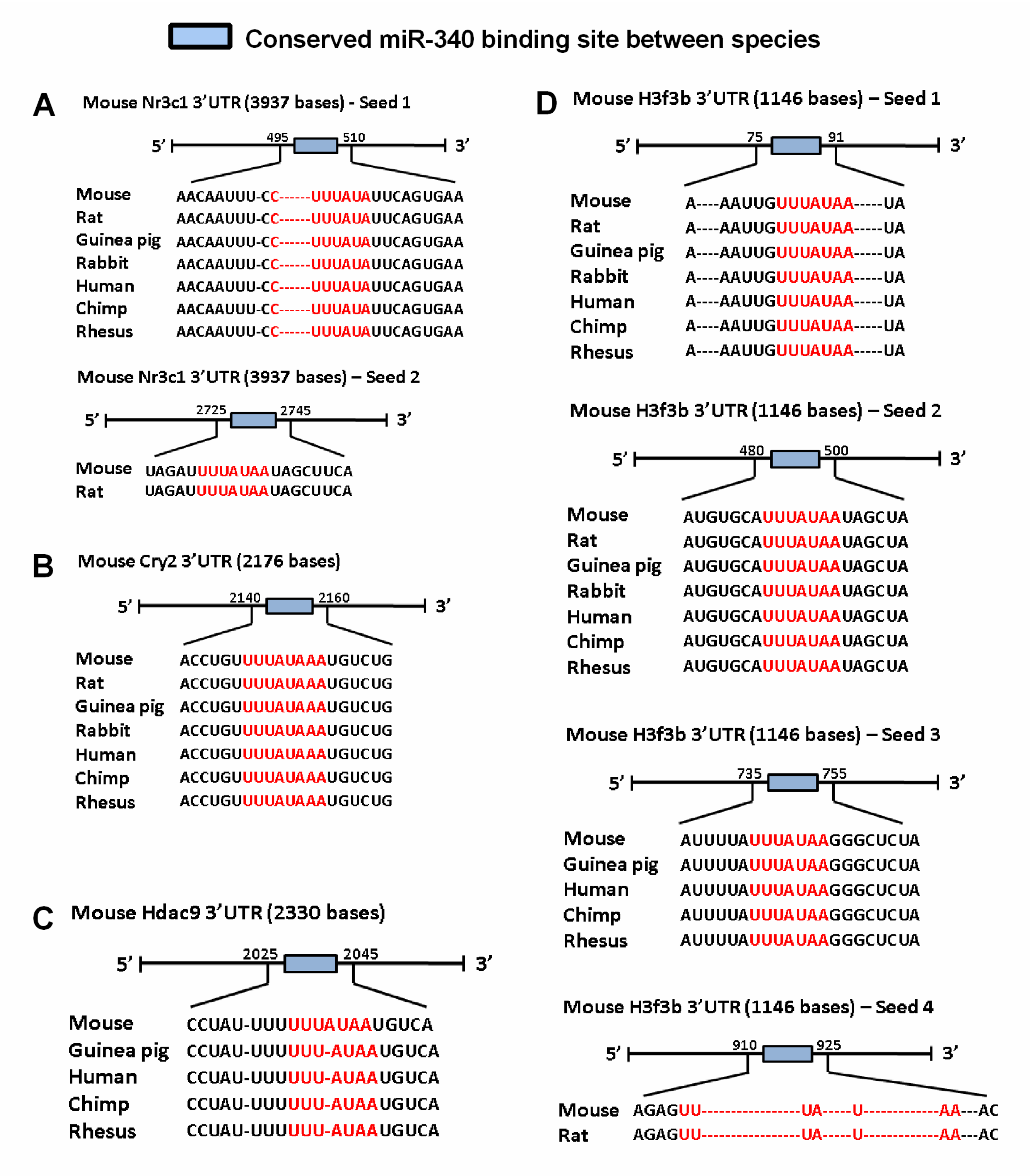

Supplementary Fig. 4 (A) Mouse Nr3c1 (GR) 3'UTR with conserved (Seed 1) and not conserved (Seed 2) seeds for miR-340. **(B)** Mouse Cry2 3'UTR with conserved seed for miR-340. **(C)** Mouse Hdac9 3'UTR with conserved seed for miR-340. **(D)** Mouse H3f3b 3'UTR with conserved (Seeds 1-3) and not conserved (Seed 4) seeds for miR-340.

Supplementary Fig. 5

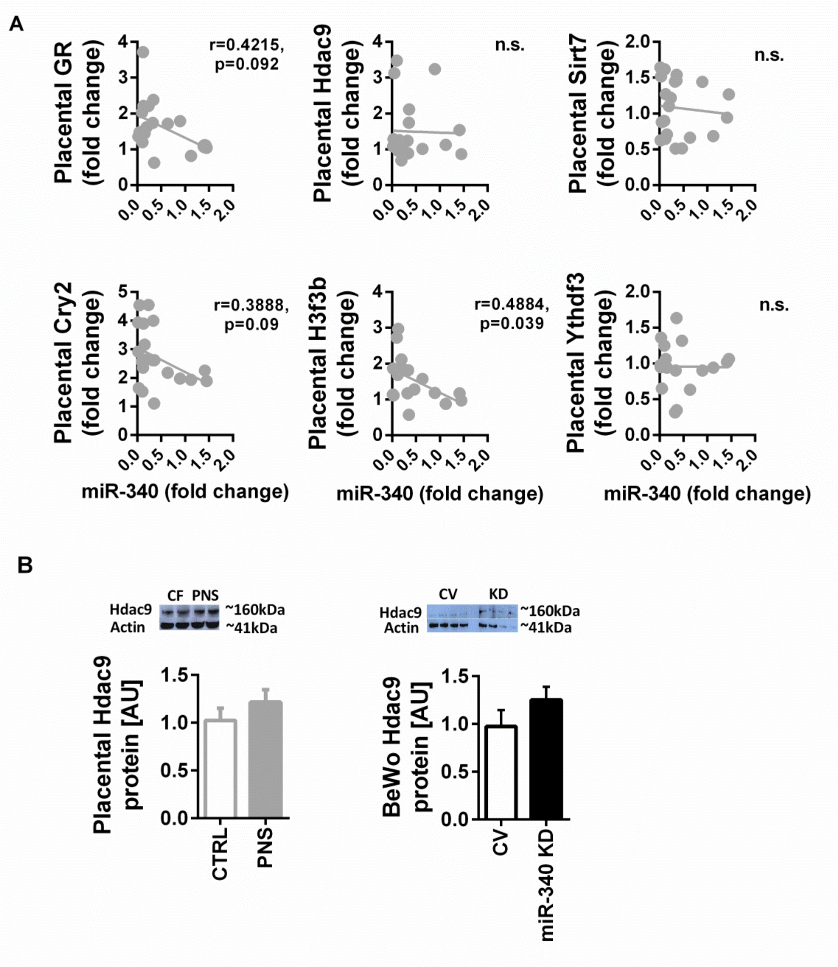

Supplementary Fig. 5 Within the PNS group, miR-340 showed a tendency to an inverse correlation with the expression of *GR* and *Cry2*. This inverse correlation was significant for *H3f3b*. *Hdac9, Sirt7 and Ythdf3* did not show an inverse correlation with the levels of miR-340. N=18. (B) Placental Hdac9 protein was not increased by PNS. MiR-340 KD in BeWo cells did not increase Hdac9 protein levels. N=4. Data are presented in mean and SEM.

Supplementary Fig. 6

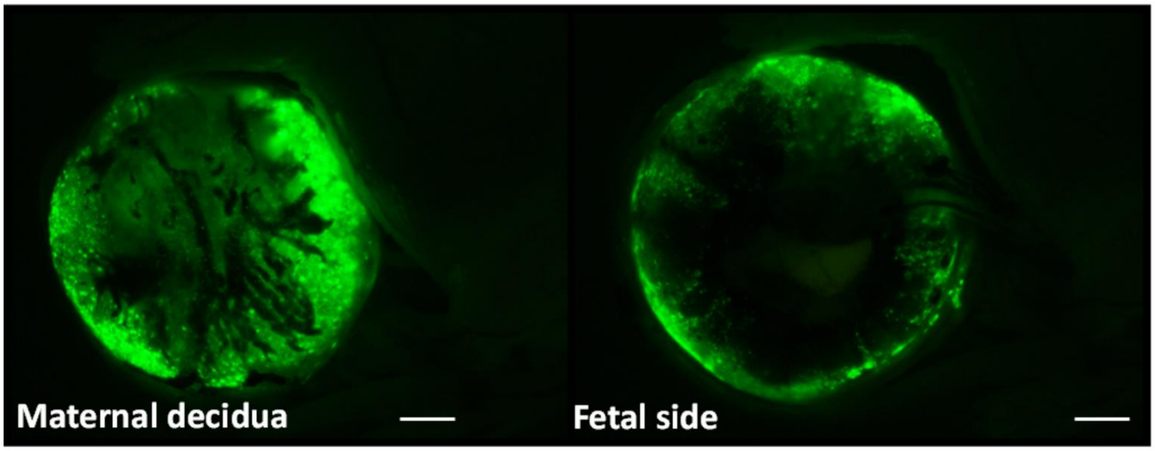

Supplementary Fig. 6 Viral miR-340 OE is relatively more concentrated in the junctional area of the placenta. In the left picture, it can be seen that the infection is weaker in the center of the top layer (maternal decidua). This is also the case for the fetal side (right picture), where GFP is not observed in the central area of the labyrinth. Scale Bar: 1mm.

Supplementary Fig. 7

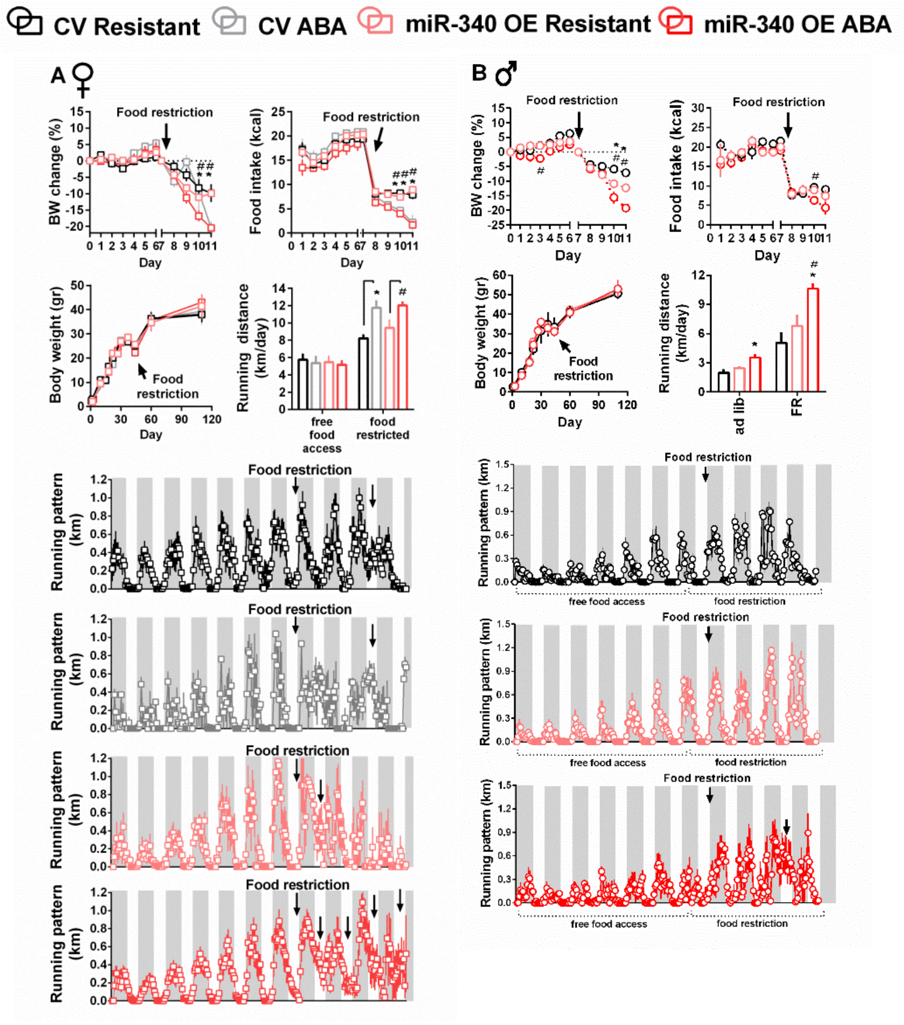

Supplementary Fig. 7 (A) Raw data parameters of the ABA protocol in female placental transgenes. The ABA protocol induced a dramatic decrease in BW ($F_{int(33,286)}$=2.628, p<0.0001) and food intake ($F_{int(30,260)}$=1.82, p=0.0073) in ABA prone compared to resistant females with both viruses (CV ABA N=4, CV Resistant N=11, miR-340 OE ABA N=11 and miR-340 OE Resistant N=4). Long term body weight was similar between the groups. Total running distance was significantly increased during FR in both ABA groups ($F_{int(3,26)}$=6.646, p=0.0018). Running pattern was normal in resistant females and disrupted in the ABA group, which displayed high activity in the light phase. *p<0.05 for CV ABA vs. CV Resistant and # p<0.05 for miR-340 OE ABA vs. miR-340 OE Resistant based on Tukey's multiple comparisons test. **(B)** Raw data parameters of the ABA protocol in male placental transgenes. The ABA protocol induced a dramatic

decrease in BW in miR-340 OE ABA prone males ($F_{int(22,144)}$=3.431, p<0.0001). No effect was observed in food intake. Long term body weight was similar between the groups. Total running distance was significantly increased during FR in the miR-340 OE ABA group ($F_{int(2,12)}$=5.579, p=0.0194). Running pattern was normal in resistant males, but was disrupted in the ABA group, which displayed high activity in the light phase. *p<0.05 for miR-340 OE ABA vs. miR-340 OE Resistant and #p<0.05 for miR-340 OE ABA vs. CV Resistant based on Tukey's multiple comparisons test. CV Resistant N=7, miR-340 OE ABA N=4 and miR-340 OE Resistant N=4. Data presented in mean and SEM.

Supplementary Fig. 8

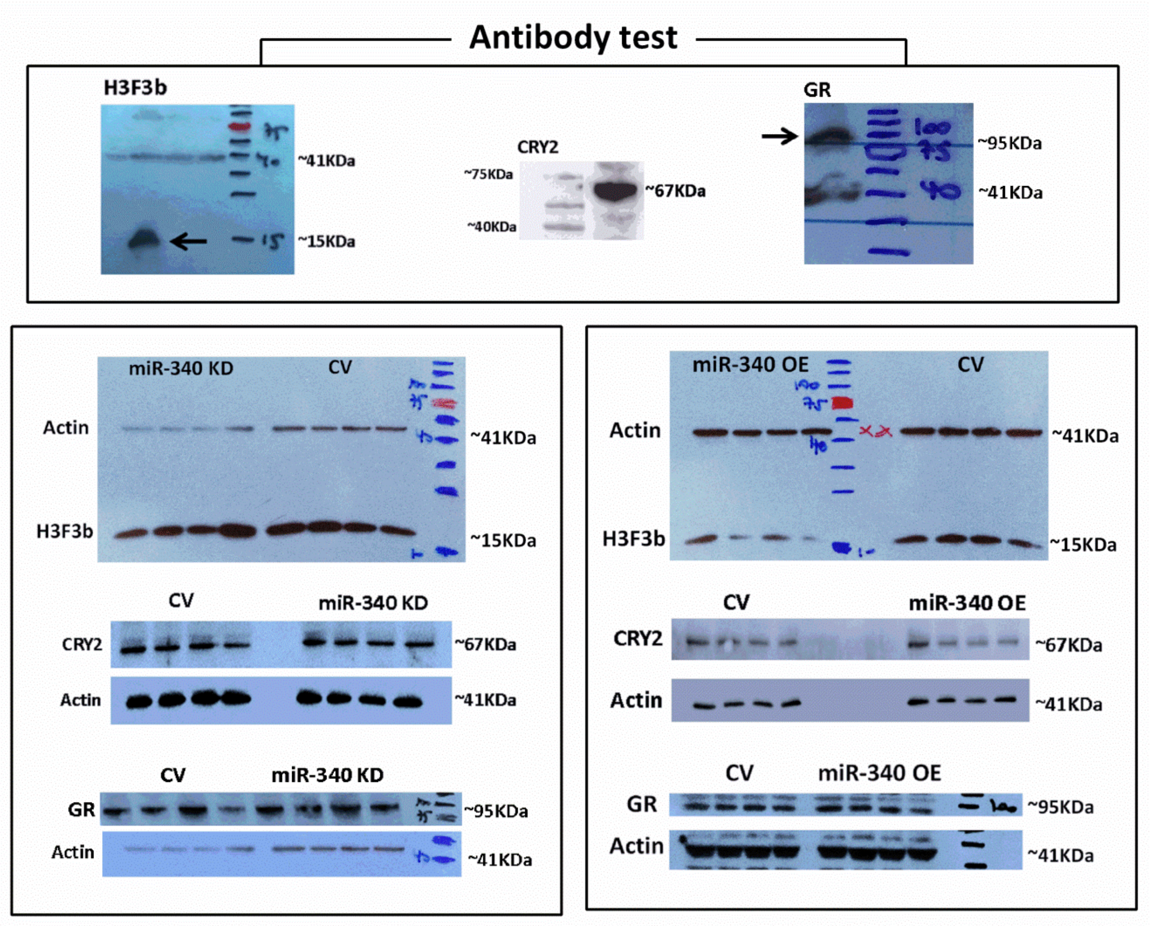

Supplementary Fig. 8 Western blot antibody tests and membranes.

Supplementary Table 1. Taqman placental gene expression predictions for miR-340 and array.

Gene	miRanda	miRDB	miRWalk	Targetscan	Conserved seeds (total)	fold change	p value
Igf2bp3	1	1	1	1	1 (1)	-1.02	0.764
**Sirt7**	**1**	**1**	**1**	**1**	**1 (1)**	**1.23**	**0.009**
Pgr	1	1	1	1	1 (1)	-1.04	0.946
**Ythdf3**	**1**	**1**	**1**	**1**	**3 (4)**	**1.21**	**0.004**
Ogt	1	1	1	1	2 (2)	1.10	0.112
Il10	1	1	1	1	1 (2)	-1.29	0.969
Robo1	1	1	1	1	1 (1)	-1.02	0.882
Rhoa	1	1	1	1	1 (1)	-1.17	0.313
Hcrtr2	1	1	1	1	1 (4)	-1.35	0.040
Mat2a	1	1	1	1	1 (1)	1.27	0.114
**Nr3c1**	**1**	**1**	**1**	**1**	**1 (2)**	**1.36**	**0.046**
Igf1	1	0	1	1	1 (1)	-1.06	0.572
Igf1r	1	0	1	1	4 (4)	-1.13	0.168
**Hdac9**	**1**	**0**	**1**	**1**	**1 (1)**	**1.54**	**0.083**
Cnrip1	1	0	1	1	1 (1)	1.14	0.365
Pparg	1	0	1	1	1 (1)	-1.01	0.916
Hif1a	1	0	1	1	1 (3)	1.29	0.145
**Cry2**	**1**	**0**	**1**	**1**	**1 (1)**	**1.94**	**0.015**
Htr2c	1	0	1	1	1 (2)	1.13	0.554
Slc2a1	1	0	1	1	1 (1)	-1.16	0.014
Slc38a4	1	0	1	1	1 (1)	-1.01	0.787
Slc38a3	1	0	1	1	1 (1)	-1.07	0.506
Pax3	1	0	1	1	1 (1)	-1.27	0.014
Mll1	1	0	1	1	1 (2)	1.04	0.548
Avpr1a	0	1	1	1	1 (2)	-1.06	0.885
Irs1	1	0	1	0	1 (2)	-1.15	0.405
**H3f3b**	**1**	**0**	**1**	**0**	**3 (4)**	**1.32**	**0.002**
Dnmt3a	1	0	1	0	1 (2)	-1.02	0.814
Alkbh5	0	1	1	0	1 (3)	1.09	0.300

Supplementary Table 2. Sample classification and clinical characteristics of patients

Term placentae N=35	Mean ± SD
Maternal age, y	32.7± 4.9
Gravidity	2.2 ± 0.9
Parity	1.9 ± 0.8
Gestational age, weeks	39.3 ± 0.5
Placental weight, g	595.1 ± 132.1
Birth weight, g	3321.3 ±448.6
Caucasian	94.3 % (33/35)
Sex of the infant, female/male	16/19

Term control: Delivery by elective caesarean section ≥ 37 gestational weeks without clinical or histopathologic abnormalities. The indication for caesarean section was either patient's wish or history of ≥ two caesarean sections. Data presented in mean ± SD.

Supplementary Table 3. Correlation between nutrient transporters and miR-340 expression in female mouse and human placentas (N=12 for mice and N=35 for human samples).

Gene name	Predominant substrate	Mouse placenta	Human placenta
Slc19a2	Thiamine (B1)	↑ p<0.05	n.s.
Slc46a1	Folate (B6)	n.s.	↓ p<0.05
Slc5a6	Biotin	↑ p<0.05	n.s.
Lmbrd1	Cobalamin (B12)	↑ p<0.05	n.s.
Slc22a15	Pyridoxine (B6)	↓ p<0.05	n.s.
Slc23a3	L-ascorbic acid (Vitamin C)	n.s.	n.s.
Slc30a4	Zinc	↑ p<0.05	n.s.
Slc26a2	Sulfate ions	↑ p<0.05	↓ p<0.05
Slc11a2	Zinc, Iron	↓ p<0.05	n.s.
Abca1	Cholesterol, phospholipids	n.s.	↓ p<0.05
Abcg1	Cholesterol, phospholipids	↑ p<0.05	↓ p<0.05
Slc2a1	Glucose	n.s.	↓ p<0.05

↑ positive correlation, ↓ negative correlation.

Supplementary Table 4. Mouse RT-PCR primers

	5'	3'
miR-340-5p	TTATAAAGCAATGAGACTGATT	miScript universal primer
U6	GATGACACGCAAATTCGTGAA	miScript universal primer
11Hsdβ2	AACCTCTGGCAGAAACGCAAG	GGCATCTACAACTGGGCTAAGG
Rnf130	CCCATCCACGGAGTTGCT	GAACCGTGTTTGAGGATCACAG
Nr3c1/GR	TGCTGTTTATCTCCACTGAATTAC	TCCTTAGGAACTGAGGAGAGAAGC
Cry2	ATGTGTTCCCAAGGCTGTTC	GGTTTCTGCCCATTCAGTTC
Sirt7	GTTTGCATGAGCAAAAGCTG	ATGCAGGAGGTGCAGACTTC
Hdac9	GCAGCAGATCCACATGAACA	AGAGGCTGCTCTGTCTTCCA
H3f3b	GGTGCCCTTCAGGAGGCTA	TGATGGTGACTCTCTTGGCG
Ythdf3	TGCACATTATGAAAAGCGTCA	AGATGCGCTGATGAAAACCA
Tbp	CTCAGTTACAGGTGGCAGCA	ACCAACAATCACCAACAGCA
AgRP	AAGCTTTGGCGGAGGTGCTAGAT	AAGCAGGACTCGTGCAGCCTTACA
Htr1a	GTGCACCATCAGCAAGGACC	GCGCCGAAAGTGGAGTAGAT
Bdnf	AGGCACTGGAACTCGCAA	AATCGCCAGCCAATTCTC
Mc4R	CAAGAACCTGCACTCACCCA	GACCCATTCGAAACGCTCAC
Hcrt	ACCACTGCACTGAAGAGATCA	CCCAGGGAACCTTTGTAGAAGGA
Avp	TCAACACTACGCTCTCCGCTT	CCTTTGCCGCCCGG
Crf	GCAGTTAGCTCAGCAAGCTCAC	CAAATGATATCGGAGCTGCG
CrfR1	TGCCAGGAGATTCTCAACGAA	AAAGCCGAGATGAGGTTCCAG
CrfR2	TACCGAATCGCCCTCATTGT	CCACGCGATGTTTCTCAGAAT
Hprt	GCAGTACAGCCCCAAAATGG	GGTCCTTTTCACCAGCAAGCT

Supplementary Table 5. Human RT-PCR primers

	5'	3'
ABCA1	CACATTTTTGCCTGGGACG	AGCGATTCTCCCCAAACCTT
ABCG1	AACATGGAGGCCACTGAGAC	GGCCACCAACTCACCACTAT
LMBRD1	CCTCTTACTACTGGCTATT	TTATGGTGGAGACAACTT
SLC2A1	GAACTCTTCAGCCAGGGTCC	ACCACACAGTTGCTCCACAT
SLC5A6	GTCCTGTACTTTGTGATG	AGGATATAGTGCTGAGAG
SLC7A5	CAGGGCATCTTCTCCACGAC	GAAAGGGCAACCATGAAGAGG
SLC7A11	TGCTGGGCTGATTTATCTTCG	TGGGTTCGAGGAGGTGATCTA
SLC11A2	TTGCGGAGCTGGTAAGAATCA	AAGACTGGCAGACTCCCCAT
SLC19A2	TCACGATAGCAACTTTTC	GGCATCTACCACAATTAG
SLC22A15	CTGGCTTGTCTTATTGTAA	CAACATTCCTGATGACTG
SLC23A3	CTGGCTTGTCTTATTGTAA	AGACAGTTCCGAAAGGAG
SLC26A2	GATTGGTGAGACAGTTGAC	CCCATCGCTACCTGATAA
SLC30A4	ACTAGAAGGTGTGCCAAGCC	AGCTGTATGTGAACTATGGCAGT
SLC38A1	CAGCGCCGGTGCATGTCGA	TGGAAGCTTGACACCCCTGTTAGC
SLC46A1	ACTCCAAACTAATCGGCTAT	AACCCATATCCTGTGAACA
SLC16A3	GCCCCCTTCGGGAGGCAAAC	GGCCCCCGCCAGGATGAAC
IGF2	GTGCTACCCCCGCCAAGT	TGGACTGCTTCCAGGTGTCA
IGF2BP1	CCTGCTGGCTCAGTATGGT	GACATTCACCACTGCCGTCTC
UBQ	TCGCAGCCGGGATTTG	GCATTGTCAAGTGACGATCACA
YWHAZ	CCGTTACTTGGCTGAGGTTG	AGTTAAGGGCCAGACCCAGT
