# Supplementary material for: Placental miR-340 mediates vulnerability to activity based anorexia in mice
Source: Nat Commun. 2018 Apr 23;9:1596. doi: 10.1038/s41467-018-03836-2 (PMC5913294; doi:10.1038/s41467-018-03836-2)
Supplement: Supplementary file 3 — Description of Additional Supplementary Files [file 41467_2018_3836_MOESM3_ESM.pdf]

Description of Additional Supplementary Files

File Name: Supplementary Data 1
Description: **Placental miR array comparing CTRL and PNS females.**
